# Variation in Acetyl-CoA Carboxylase Beta Gene and Its Effect on Carcass and Meat Traits in Gannan Yaks

**DOI:** 10.3390/ijms242015488

**Published:** 2023-10-23

**Authors:** Chune Zhu, Youpeng Qi, Xiangyan Wang, Baohong Mi, Changze Cui, Shaopeng Chen, Zhidong Zhao, Fangfang Zhao, Xiu Liu, Jiqing Wang, Bingang Shi, Jiang Hu

**Affiliations:** Gansu Key Laboratory of Herbivorous Animal Biotechnology, College of Animal Science and Technology, Gansu Agricultural University, Lanzhou 730070, China; 18394174334@163.com (C.Z.); qiyp_gsau@163.com (Y.Q.); wxy9242022@163.com (X.W.); mi_baohong@163.com (B.M.); cuichangze0120@163.com (C.C.); 15095373670@163.com (S.C.); zhaozd@gsau.edu.cn (Z.Z.); zhaofangfang@gsau.edu.cn (F.Z.); liuxiu@gsau.edu.cn (X.L.); wangjq@gsau.edu.cn (J.W.)

**Keywords:** *ACACB*, SNPs, haplotype, carcass traits, meat quality traits, yak

## Abstract

Acetyl-CoA carboxylase beta (*ACACB*) is a functional candidate gene that impacts fat deposition. In the present study, we sequenced exon 37–intron 37, exon 46–intron 46, and intron 47 of yak *ACACB* using hybrid pool sequencing to search for variants and genotyped the gene in 593 Gannan yaks via Kompetitive allele-specific polymerase chain (KASP) reaction to determine the effect of *ACACB* variants on carcass and meat quality traits. Seven single nucleotide polymorphisms were detected in three regions. Eight effective haplotypes and ten diplotypes were constructed. Among them, a missense variation g.50421 A > G was identified in exon 37 of *ACACB*, resulting in an amino acid shift from serine to glycine. Correlation analysis revealed that this variation was associated with the cooking loss rate and yak carcass weight (*p* = 0.024 and 0.012, respectively). The presence of haplotypes H5 and H6 decreased Warner–Bratzler shear force (*p* = 0.049 and 0.006, respectively), whereas that of haplotypes H3 and H4 increased cooking loss rate and eye muscle area (*p* = 0.004 and 0.034, respectively). Moreover, the presence of haplotype H8 decreased the drip loss rate (*p* = 0.019). The presence of one and two copies of haplotypes H1 and H8 decreased the drip loss rate (*p* = 0.028 and 0.004, respectively). However, haplotype H1 did not decrease hot carcass weight (*p* = 0.011), whereas H3 increased the cooking loss rate (*p* = 0.007). The presence of one and two copies of haplotype H6 decreased Warner–Bratzler shear force (*p* = 0.014). The findings of the present study suggest that genetic variations in *ACACB* can be a preferable biomarker for improving yak meat quality.

## 1. Introduction

Yaks (*Bos grunniens*) are unique, large ruminant domestic animals found in the Qinghai–Tibet Plateau and surrounding high altitudes; they can survive in extremely cold, hypoxic, and other harsh climatic conditions [1]. Yaks steadily provide meat, milk, and yak wool fiber to local herders over a long period [2]. Gannan yaks are distributed in highlands (>2800 m above sea level) in the Gannan region of Gansu Province, northwestern China. These yaks grow on natural and organic green pastures and feed on them [3]; therefore, their products are called “green food”.

Yak meat, a special livestock product of the highland region, provides important animal protein for the diet of local herders [4,5]. Compared with common beef, yak meat is richer in calcium, phosphorus, and other trace elements, with high protein content and nutritional value and low fat content [6]. The two important edible sensory qualities of beef that affect consumer acceptance are tenderness and intramuscular fat (IMF) content. However, yak meat has thicker muscle fibers and less IMF deposition. At the consumer level, recently, more consumers have been willing to buy beef of better quality. At present, variation in beef tenderness is an important issue faced by the beef industry and is a critical factor affecting its economy [7].

The development of basic meat composition and quality is affected by various factors, including genetic, nutritional, physiological, and environmental factors [8]; of them, genetic factors are the main determinants [9]. Fat and fatty acid composition is closely associated with the flavor, appearance, texture, and hardness of meat [10,11,12,13]. The degree of marbling is defined as the number and distribution of IMF—the main determinant of beef quality. It has a positive correlation with tenderness, juiciness, and flavor [14]. Furthermore, the main quality traits that should be controlled in beef are tenderness and IMF; these are crucial to the livestock economy [15,16]. However, owing to the special natural environment and traditional concepts, the Chinese yak industry has inefficient production. Therefore, exploring the development of high-quality yak meat is vital. Previous studies have reported that most meat quality traits, particularly meat tenderness, exhibit genetic differences in cattle herds [17,18,19,20]. Many genes related to adipogenesis and metabolism are directly or indirectly associated with IMF content [21].

Acetyl-CoA carboxylase (ACC) was discovered by Salih and coworkers in the late 1950s. It is a biotin-dependent enzyme that catalyzes the conversion of acetyl-CoA to malonyl-CoA during fatty acid biosynthesis and regulates fat deposition by participating in metabolic processes [22,23]. Acetyl-CoA carboxylase is derived from the acyl-CoA superfamily, including *ACACA*, *ACAT1*, *ACAA2*, *ACACB*, *ACADS*, and *ACADVL*. It is a rate-limiting enzyme in fatty acid oxidation that plays an important catalytic role in fatty acid synthesis and β-oxidation. It comprises two isoforms: ACC-α and ACC-β, which are encoded by *ACACA* and *ACACB*, respectively [24]. The acetyl-CoA carboxylase beta gene catalyzes the carboxylation of acetyl-CoA to malonyl-CoA, a major precursor of fatty acid synthesis. However, when *ACACB* expression increases, fatty acid oxidation can be controlled by inhibiting the activity of carnitine palmitoyltransferase 1 (CPT-1) [25]. Among the various major factors contributing to fat deposition, free fatty acid accumulation is extremely critical. The acetyl-CoA carboxylase beta gene is involved in fatty acid metabolism and is mainly expressed in the heart and liver, as well as in skeletal muscle [26]. Acetyl-CoA carboxylase beta gene knockout mice have sustained fatty acid oxidation in the adipocytes, conferring protective effects against obesity and diabetes [27]. Furthermore, in women with kidney disease and postmenopausal women, *ACACB* variants are associated with obesity and type II diabetes [28,29]. Moreover, in Alentejana bulls, *ACACB* is associated with the IMF content and fatty acid composition of beef [30]. The variation of 368 C/T in human *ACACB* genes affects the promoter activity in an allele-specific fashion [31]. Therefore, *ACACB* is variable and may play a vital role in the regulation of IMF and obesity.

Decreasing fat deposition can increase the economic value of meat and improve feeding efficiency. However, most of the present studies on *ACACB* have focused on cancer and some obesity-related diseases, and only a few studies have focused on beef quality traits. Therefore, in the present study, we used *ACACB* as an entry point to screen genetic variants in the yak population and determined the correlation between *ACACB* variations and the meat quality traits of Gannan yaks. The study’s findings may provide a theoretical basis for molecular genetic studies on the meat quality traits of yaks.

## 2. Results

### 2.1. Subsection

#### 2.1.1. Identification of Sequence Variation in Yak *ACACB*

**Figure 1:**
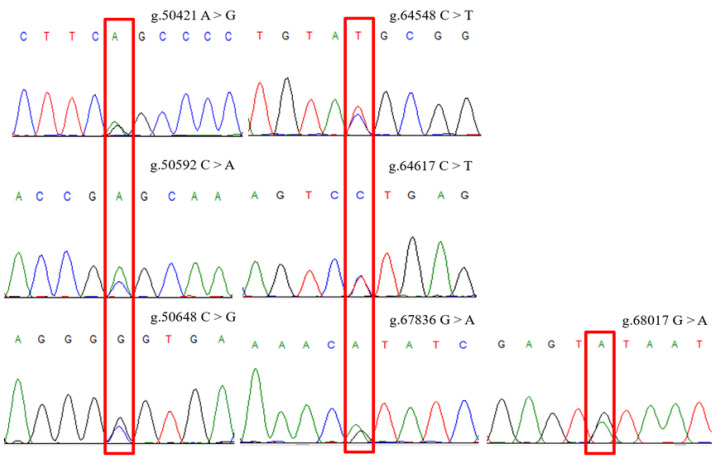
Polymerase chain reaction (PCR) amplification and sequencing results of *ACACB* in Gannan yaks. The overlapping peak indicates the single nucleotide polymorphisms (SNPs).

**Figure 2:**
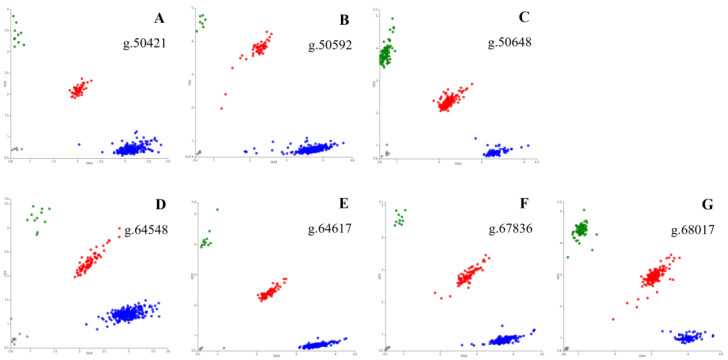
Kompetitive allele-specific PCR (KASP) genotyping assay results of seven positions of *ACACB* in Gannan yaks. The red, blue, and green dots in (**A**,**F**) and (**G**) indicate the GA, GG, and AA genotypes, respectively; those in (**B**) indicate the AC, CC, and AA genotypes, respectively; those in (**C**) indicate the GC, GG, and CC genotypes, respectively; those in (**D**,**E**) indicate the CT, CC, and TT genotypes, respectively.

The genomic DNA of 20 Gannan yaks was used to amplify the exon 37–intron 37, exon 46–intron 46, and intron 47 regions of *ACACB*, followed by the sequencing of all amplicons. Seven novel SNPs were identified at g.50421 A > G, g.50592 C > A, g.50648 C > G, g.64548 C > T, g.64617 C > T, g.67836 G > A, and g.68017 G > A (Figure 1). The locations of SNPs on the chromosomes are chr17:1393930 A > G, chr17:1393764 C > A, chr17:1393706 C > G, chr17:1379806 C > T, chr17:1379741 C > T, chr17:1376519 G > A, and chr17:1376338 G > A, respectively. The SNPs were genotyped using KASP; all SNPs had three genotypes (Figure 2). The genotype frequency of GG and allele frequency of G were the highest at position g.50421, and nucleotide transition from A to G led to an amino acid change from serine to glycine. Moreover, the genotype frequency of CC and allele frequency of C were the highest at position g.50592, genotype frequency of GC and allele frequency of G were the highest at position g.50648, genotype frequency of CC and allele frequency of C were the highest at positions g.64548 and g.64617, genotype frequency of GG and allele frequency of G were the highest at position g.67836, and genotype frequency of AG and allele frequency of A were the highest at position g.68017.

The population genetic analysis of the seven positions in Gannan yaks revealed that g.50648 and g.68017 were moderately polymorphic (0.25 < PIC < 0.5) and g.50421, g.50592, g.64548, g.64617, and g.67836 were low polymorphic (PIC < 0.25). The seven positions were in HWE in yak population (*p* > 0.05) (Table 1).

Next, linkage disequilibrium and haplotype analyses revealed that the SNPs were in a weak linkage state (r^2^ < 0.33), except for a strong linkage state between g.50421 and g.50592 (r^2^ = 0.92) (Table 2). D’ and r^2^ are two common parameters that represent linkage disequilibrium. The D’ value reflects the probability of recombination events in the linkage disequilibrium region, whereas the r^2^ value is associated with the effectiveness of linkage analysis. r^2^ considers the effects of recombination and mutation rates, which can more objectively reflect the linkage disequilibrium between different positions. Therefore, in the present study, r^2^ was used for linkage disequilibrium. Haplotypes were inferred from the genotype data according to the principle of combining alleles at multiple loci that are co-inherited on the same chromosome using the online software SHEsis (SHEsisPlus Online Version—Beta: http://shesisplus.bio-x.cn/SHEsis.html, accessed on 27 December 2022) [32,33,34,35]. Eight haplotypes with frequencies greater than 0.03 were constructed in the tested Gannan yak population. These haplotypes formed 10 diplotypes with frequencies greater than 0.03 (Table 3).

#### 2.1.2. Association between Yak *ACACB* Genotype and Carcass and Meat Quality Traits

Individuals with the GA genotype at position g.50421 had a higher cooking loss rate (CLR; %) than those with the GG genotype (*p* < 0.05). On the other hand, they had a lower hot carcass weight (HCW; kg) than those with the AA genotype (*p* < 0.05). Furthermore, individuals with the AA genotype at position g.50592 had a higher CLR than those with the AC and CC genotypes (*p* < 0.05). In addition, individuals with the GC genotype at position g.50648 had a higher drip loss rate (DLR; %) than those with the CC and GG genotypes (*p* < 0.05). Individuals with the CT genotype at position g.64548 had a higher Warner–Bratzler shear force (WBSF; kg) than those with the CC and TT genotypes; in contrast, they had a lower DLR than those with the TT genotype (*p* < 0.05). Individuals with the AA genotype at position g.67836 had a significantly higher DLR than those with the GA and GG genotypes (*p* < 0.01) (Table 4).

#### 2.1.3. Association between Yak *ACACB* Haplotype and Carcass and Meat Quality Traits

Table 5 presents the associations between the *ACACB* haplotypes and carcass and meat quality traits. In the single-haplotype (presence or absence) models, the presence of haplotypes H5 and H6 was associated with decreased WBSF (*p* = 0.049 and 0.006, respectively), whereas the presence of haplotype H3 and H4 was associated with an increased CLR (*p* = 0.004) and rib eye area (REA; cm^2^) (*p* = 0.034), respectively. Furthermore, the presence of haplotype H8 was associated with decreased DLR (*p* = 0.019). When other haplotypes (*p* < 0.2) were included in the models, their associations were significant (*p* < 0.05). No haplotypes had an association with HCW in Gannan yak *ACACB* (*p* > 0.05).

A second set of analyses was performed using the copy number of the haplotype present (presence/absence, Table 6). The presence of one and two copies of haplotypes H1 and H8 was associated with decreased DLR (*p* = 0.028 and 0.004, respectively). Furthermore, compared with noncarriers in the single-haplotype and multi-haplotype models, H1 was associated with decreased HCW (*p* = 0.011), whereas H3 was associated with increased CLR (*p* = 0.007). For WBSF, the presence of two copies of H6 was associated with decreased WBSF (*p* = 0.014), and the presence of H5 was associated with decreased WBSF (*p* = 0.075). The association of these haplotypes remained significant (*p* < 0.2) when other haplotypes were included in the models. The presence of H5 was significantly associated with decreased WBSF (*p* = 0.046). Nevertheless, no associations were observed between the copy numbers of these haplotypes and REA in Gannan yak *ACACB*.

Furthermore, we did not observe the significant effect of any diplotypes on carcass and meat quality traits. However, H5H6 had a significant tendency to increase HCW compared with other diplotypes (*p* = 0.095) (Table 7).

## 3. Discussion

This is the first study to determine the relationship between sequence variation in *ACACB* and the carcass and meat quality traits of yaks. The results suggest the presence of genetic variations in yak *ACACB* that have a significant effect on the tenderness of Gannan yaks. This confirms the variability of *ACACB* in yaks and suggests that further research on *ACACB* gene variation in yak breeds is of interest.

Because *ACACB* synthesizes malonyl-CoA for CPT-1 inhibition, it can be an attractive candidate gene for disorders of energy metabolism, including obesity and diabetes, that can primarily regulate impaired fatty acid oxidation. Studies on fatty acid oxidation have reported that the allelic variants of human *ACACB* may be associated with metabolic syndrome [36]. Furthermore, *ACACB* regulates fatty acid oxidation in the skeletal muscle, liver, and heart [37], and its variants are associated with lipid metabolism [29,38]. Acetyl-CoA carboxylase beta gene polymorphism (rs2268388, G > A) is associated with diabetes, diabetic nephropathy, insulin resistance, and obesity in some human populations [29,39,40,41]. Acetyl-CoA carboxylase beta gene SNPs and their haplotypes, which are associated with milk fat traits in Chinese Holstein cows, were identified, and it was concluded that *ACACB* variation impacts fatty acid metabolism and regulates fat mobilization, which ultimately affects milk quality [42]. Taken together, these studies suggest that the variability of *ACACB* regulates obesity and milk quality via fatty acid oxidation. Nevertheless, further studies are warranted to elucidate the relevance of *ACACB* as it relates to fat. At present, the content and composition of fatty acids in meat products are being extensively evaluated both at the national and international levels with the primary aim of producing better-quality meat products.

In the present study, seven SNPs were detected in the three fragments of Gannan yak *ACACB* examined. Among them, the variant sites g.50592, g.50648, g.64617, g.67836, and g.68017 were located in introns. Genotype association analysis revealed that mutations in the intron regions were significantly correlated with Gannan yak meat quality in terms of tenderness, leanness, water loss, eye muscle area, and carcass weight. Some previous studies have reported that genetic variation in the intron regions plays an important role in regulating their transcription, translation, and biological functions [43,44]. This genetic variation is most likely in LD with causative variation. It has been reported that the rs2075786 SNP in *TERT* is associated with a differential risk of developing cancer for *MSH2* pathogenic variant carriers [45]. McNamara et al. have reported that the *TNNT2* intronic mutation is the most likely cause of this case of feline cardiomyopathy [46]. In many eukaryotes, SNPs in introns affect gene expression by regulating transcription and translation. Furthermore, the presence of one or more introns is needed for the optimal expression of several genes [47]. Although intronic variations may not directly affect the structure of a gene [48,49], they can affect transcriptional efficiency by affecting regulatory elements such as enhancers, silencers, or other DNA structures [49]. Gao et al. have reported that variants in introns 1 and 15 of yak *DGAT1* were positively correlated with yak meat tenderness [50]. Furthermore, Angiolillo et al. have reported that variants in intron 16 of *DGAT1* are correlated with milk fat content in goats [51]. Wang et al. have reported that the g.112558859 A > G motif in intron 1 of *LCORL* can be used as a potential candidate marker, affecting body size and rawhide weight [52]. In addition, Wang et al. have reported that polymorphisms in intron 8 of *OBR* are associated with obesity traits such as abdominal fat in chickens [53]. Grochowska et al. have reported that polymorphisms in intron 1 of *MSTN* exert a significant effect on body weight and loin and anterior calf weight in colored Polish Merino sheep [54]. The results of the present study are generally consistent with those of previous studies. This suggests that the variation of the intronic region may affect gene expression and some economic traits in yaks. Nevertheless, all coding region polymorphisms within the *ACACB* have not been identified in this study, and it is possible that these non-coding region polymorphisms are in LD with a causative variant.

In the present study, a missense mutation, g.50421 A > G, was identified in exon 37 of *ACACB* in Gannan yaks. Correlation analysis revealed that this variant was significantly associated with the CLR and HCW of yaks. The codon serine is mutated to glycine. Glycine is not only a protein component but also a bioactive amino acid involved in gene expression regulation [55]. The addition of glycine to the diet can regulate the carcass trait and meat quality of Huanjiang mini-pigs [56]. This nonsynonymous variation may affect protein structure, thereby affecting phenotypic function; this is consistent with our findings. Nevertheless, the relationship between genotype and economic yak traits should be further explored and verified.

Haplotype information comprises multiple markers and plays an important role in many cases, including linkage analysis, association studies, and population genetics [57]. Genomic selection enhanced using QTL information facilitates faster genetic gain in low heritability traits [58]. In general, association analysis between haplotypes and SNPs can help accurately identify molecular marker results [59]. Haplotype analysis provides richer information and more accurate statistical results and is more effective than single SNP analysis in transmitting haplotypes [60]. Hu et al. studied Gannan yaks and reported that the presence or absence of *ANK1* haplotypes and haplotype copy number affect carcass weight, muscle water loss, and shear force [6]. In addition, An et al. reported that the absence of haplotype A2-B5 in *UCP1* in New Zealand Romney lambs is associated with an increase in HCW and loin lean meat yield [61]. Han et al. studied *ACACB* in Chinese Holstein cows and reported that haplotypes were significantly associated with milk yield and composition [42]. In the present study, the presence of haplotypes H1 and H8 was associated with decreased DLR, that of haplotype H1 was associated with decreased HCW, that of haplotype H3 was associated with increased CLR, and that of haplotype H6 was associated with decreased WBSF. We hypothesize that yak *ACACB* variation affects the drip loss rate and CLR of yak meat. We constructed ten diplotypes with frequencies greater than 0.03. Association analysis with carcass and meat quality traits revealed that the diplotype H5H6 was associated with increased HCW (*p* = 0.095); this was a significant trend compared with other diplotypes. Haplotypes are sequences of genetic variation that occur together along a single chromosome. The results of this study suggest that yak *ACACB* variations affect the WBSF, CLR, DLR, and HCW of meat. However, a limitation of this study is that only some Gannan yaks were investigated. Therefore, it remains unclear whether these results can be determined and extended to other cattle breeds. Yaks from different farms, a higher number of yak samples, and yak breeds will be investigated in future studies to further confirm the results of the present study as well as to discover more valuable and new variants. These newly identified variants have significant effects on the meat quality and carcass weight of yaks, suggesting the importance of studying yak *ACACB* variations.

## 4. Materials and Methods

### 4.1. Animals and Sample Collection

All animal experiments were carried out in accordance with the guidelines from the Gansu Agricultural University Animal Care Committee (2006-398).

During the study, at slaughter, blood samples were collected from the jugular vein of 593 Gannan yaks, stored in (Acid-Citrate-Dextrose, ACD) anticoagulant tubes, and stored at −70 °C. These yaks were raised in the same feeding environment and management conditions in the Gannan Tibetan Autonomous Prefecture, Gansu Province, China. We recorded the sex, age, and population of each yak. Genomic DNA for PCR amplification was isolated using a TIANamp Blood DNA Kit (TIANGEN) according to the manufacturer’s instructions [42].

### 4.2. Measurement of Carcass and Meat Quality Traits

After slaughter, the HCW of 593 Gannan yaks was determined and blood samples were collected. Forty-eight hours after slaughter, the REA of each animal was measured using sulfate paper and estimated using a grid. Then, a portion of the *Longissimus* muscle from the 12th to 13th ribs of the right carcass side was packaged, quickly frozen, and stored at −18 °C for quality assessment. The WBSF, which represents meat tenderness, was measured using a digital muscle tenderometer (C-LM3, Northeast Agricultural University, Harbin, China). DLR and CLR were determined using the methods described by Liu et al. [62] and Honikel [63], respectively.

### 4.3. Polymerase Chain Reaction (PCR) Amplification and Genotyping

Three PCR primer pairs were designed using Primer 5.0 (Table 8) to amplify three regions (exon 37–intron 37, exon 46–intron 46, and intron 47) of *ACACB* using a wild yak *ACACB* sequence (GenBank No. NW_005393292.1). The primers were synthesized by TsingKe Biotechnology Co., Ltd. (Xi’an, China). The total PCR amplification reaction volume was 20 µL: 0.8 µL of genomic DNA, 0.8 µL of each primer, 10.0 µL of Taq DNA polymerase, and 7.6 µL of ddH_2_O. The cycling conditions were as follows: 2 min at 94 °C, followed by 35 cycles of 30 s at 94 °C, 30 s at the annealing temperatures (Table 1), and 30 s at 72 °C, followed by a final extension of 5 min at 72 °C. PCR amplification products were sequenced by Sangon Biotech Co., Ltd. (Shanghai, China). The sequencing results were detected using DNAMAN (version 5.2.10, Lynnon BioSoft, Vaudreuil, QC, Canada) to detect SNPs. Kompetitive allele-specific PCR (KASP) genotyping assays were performed by Gentides Biotech Co., Ltd. (Wuhan, China). The fluorescence data were collected by employing a microplate reader with a fluorescence resonance energy transfer (FRET) probe. Genotyping maps were created using the online software (http://www.snpway.com/snpdecoder/, accessed on 24 August 2022). LGC-OMEGA software.

### 4.4. Statistical Analyses

Genotype frequencies, allele frequencies, Hardy–Weinberg equilibrium (HWE), polymorphism information content (PIC), homozygosity, heterozygosity, and the effective allele numbers of the SNPs of *ACACB* were calculated using Microsoft Excel 2016. Linkage disequilibrium (LD) and haplotype analysis of SNPs were performed using the online software SHEsis (http://shesisplus.bio-x.cn/SHEsis.html, accessed on 27 December 2022).

The associations between the different genotypes and haplotypes (frequencies of >3%) and values of carcass and meat quality traits of yak were determined using the general linear mixed models (GLMMs) of IBM SPSS 26.0 software (IBM Corp., Armonk, NY, USA). The model was calculated as follows: Y_ijkl_ = µ + G_i_ (H_i_) + P_j_ + S_k_ + A_l_ + e_ijkl_, where Y_ijkl_ represents the phenotypic observation, µ represents the population mean, G_i_ or H_i_ represent the fixed effect of the genotype or the fixed effect of the *i*^th^ haplotype (*i* = 0 or 1) or the fixed effect of the *i*^th^ number of copies of the haplotype (*i* = 0, 1, 2), P_j_ represents the fixed effect of the population, S_k_ represents the effects of sex, A_l_ represents the effects of age, and e_ijkl_ represents random error. Population, age, and sex were included in the statistical mode as fixed factors. Unless otherwise mentioned, all *p* values less than 0.05 were considered to be significantly different.

First, single-haplotype models were used to determine the presence of a relationship. All haplotypes with *p* > 0.2 potentially affected carcass and meat quality traits and were included in the multivariate model. Therefore, if the haplotypes that possibly affected traits were considered in the model, we could determine the effects of independent haplotypes.

A second set of models, similar to GLMMs, which was used to test the single haplotype (presence or absence), was constructed using the number of haplotype copies present (presence or absence).

In addition, for combined haplotypes with frequencies of >3% (providing sufficient sample size), a second set of GLMMs was used to determine the effect of combined haplotypes on carcass and meat quality traits. The Bonferroni procedure was performed for multiple comparisons if significant associations were identified in these models.

## 5. Conclusions

Seven SNPs were identified in the detection regions of yak *ACACB*. Eight haplotypes and ten combined haplotypes were constructed. Among them, different genotypes at g.50421 A > G, g.50592 C > A, g.50648 C > G, g.64548 C > T, and g.67836 G > A loci affect carcass and meat quality traits, and the presence of haplotypes H5 and H6 contributed to the improvement of tenderness, which can be used for the genetic improvement of meat quality traits through marker-assisted selection, and may be used in selection along with other traits to improve the economic value of yak. In the later studies, it is necessary to further expand the research scope of this gene and actively carry out functional validation so that it can be more accurately applied to the genetic improvement of yak populations.

## Figures and Tables

**Table 1:** Population genetics of the seven positions of yak *ACACB*.

Locus	Genotype Frequency/%			Allele Frequency/%		PIC ^1^	He ^2^	Ho ^3^	Ne ^4^	HWE ^5^
g.50421 A > G	AA(19) 3.22	GA(109) 18.47	GG(462) 78.31	*A* 12.46	*G* 87.54	0.1943	0.2181	0.7819	1.2790	*p* > 0.05
g.50592 C > A	AA(11) 1.86	AC(119) 20.17	CC(460) 77.97	*A* 11.95	*C* 88.05	0.1883	0.2104	0.7896	1.2665	*p* > 0.05
g.50648 C > G	CC(131) 22.24	GC(312) 52.97	GG(146) 24.79	*C* 48.73	*G* 51.28	0.3748	0.4997	0.5003	1.9987	*p* > 0.05
g.64548 C > T	CC(443) 75.08	CT(132) 22.37	TT(15) 2.54	*C* 86.27	*T* 13.73	0.2088	0.2368	0.7631	1.3104	*p* > 0.05
g.64617 C > T	CC(401) 68.20	CT(166) 28.23	TT(21) 3.57	*C* 82.32	*T* 17.69	0.2488	0.2912	0.7088	1.4108	*p* > 0.05
g.67836 G > A	AA(12) 2.04	GA(158) 26.87	GG(418) 71.09	*A* 15.48	*G* 84.53	0.2274	0.2616	0.7384	1.3543	*p* > 0.05
g.68017 G > A	AA(177) 29.95	GA(269) 45.52	GG(145) 24.53	*A* 52.71	*G* 47.29	0.3743	0.4985	0.5015	1.9942	*p* > 0.05

^1^ Polymorphism information content; ^2^ heterozygosity; ^3^ homozygosity; ^4^ effective allele numbers; ^5^ Hardy–Weinberg equilibrium.

**Table 2:** Linkage disequilibrium analysis of the seven single nucleotide polymorphisms (SNPs) of *ACACB*.

Locus	g.50421 A > G		g.50592 C > A		g.50648 C > G		g.64548 C > T		g.64617 C > T		g.67836 G > A		g.68017 G > A	
	D’	r^2^	D’	r^2^	D’	r^2^	D’	r^2^	D’	r^2^	D´	r^2^	D’	r^2^
g.50421 A > G	-	-	0.99	0.92	0.98	0.13	0.95	0.02	0.69	0.01	0.99	0.02	0.98	0.12
g.50592 C > A	-	-	-	-	0.98	0.12	1.00	0.02	1.00	0.02	1.00	0.02	0.98	0.11
g.50648 C > G	-	-	-	-	-	-	1.00	0.16	0.62	0.07	0.91	0.15	0.31	0.09
g.64548 C > T	-	-	-	-	-	-	-	-	1.00	0.03	0.91	0.02	0.97	0.13
g.64617 C > T	-	-	-	-	-	-	-	-	-	-	0.99	0.03	0.79	0.12
g.67836 G > A	-	-	-	-	-	-	-	-	-	-	-	-	1.00	0.20
g.68017 G > A	-	-	-	-	-	-	-	-	-	-	-	-	-	-

**Table 3:** Haplotypes and diplotypes of the seven single nucleotide polymorphisms (SNPs) of *ACACB*.

Haplotype	g.50421 A > G	g.50592 C > A	g.50648 C > G	g.64548 C > T	g.64617 C > T	g.67836 G > A	g.68017 G > A	Frequency	Diplotypes	Frequency
H1	G	C	C	C	C	G	G	0.271	H1H1	0.069
H2	G	C	G	C	T	G	A	0.093	H1H2	0.054
H3	A	A	G	C	C	G	A	0.115	H1H3	0.073
H4	G	C	G	C	C	A	G	0.138	H1H4	0.076
H5	G	C	C	T	C	G	A	0.136	H1H5	0.057
H6	G	C	G	C	C	G	A	0.088	H1H6	0.039
H7	G	C	G	C	C	G	G	0.035	H4H5	0.040
H8	G	C	C	C	T	G	A	0.045	H4H6	0.030
									H5H6	0.039
									H7H8	0.054

**Table 4:** Association between genotype and carcass and meat quality traits in yak.

Locus	Genotype	Meat Quality					Carcass Quality	
		n	WBSF (kg)	CLR (%)	DLR (%)	REA (cm^2^)	n	HCW (kg)
g.50421 A > G	AA	19	5.18 ± 0.35	67.44 ± 1.25 ^ab^	23.05 ± 1.31	32.61 ± 1.93	6	157.25 ± 13.56 ^a^
	GA	109	5.45 ± 0.16	67.57 ± 0.56 ^a^	21.28 ± 0.59	31.84 ± 0.86	39	113.78 ± 6.18 ^b^
	GG	462	5.46 ± 0.10	66.00 ± 0.37 ^b^	21.53 ± 0.39	32.36 ± 0.58	153	111.25 ± 3.83 ^b^
	*p*-value		0.836	**0.024**	0.614	0.827		**0.012**
g.50592 C > A	AA	11	5.25 ± 0.45	68.94 ± 1.63 ^a^	20.44 ± 1.71	28.58 ± 2.52	0	/
	AC	119	5.45 ± 0.15	67.31 ± 0.54 ^ab^	21.55 ± 0.56	32.22 ± 0.83	46	122.16 ± 5.86
	CC	460	5.46 ± 0.10	66.03 ± 0.37 ^b^	21.42 ± 0.39	32.28 ± 0.58	151	112.96 ± 3.89
	*p*-value		0.960	**0.040**	0.242	0.400		0.101
g.50648 C > G	CC	131	5.49 ± 0.15	66.34 ± 0.53	20.51 ± 0.55 ^b^	31.79 ± 0.81	47	113.30 ± 5.71
	GC	312	5.45 ± 0.11	66.28 ± 0.41	21.81 ± 0.42 ^a^	32.57 ± 0.62	114	113.97 ± 4.30
	GG	146	5.48 ± 0.15	66.74 ± 0.54	21.73 ± 0.56 ^ab^	31.98 ± 0.83	36	117.80 ± 6.24
	*p*-value		0.238	0.792	**0.038**	0.782		0.842
g.64548 C > T	CC	443	5.44 ± 0.10 ^ab^	66.29 ± 0.37	21.33 ± 0.38 ^b^	32.54 ± 0.57	148	112.34 ± 3.89
	CT	132	5.60 ± 0.15 ^a^	66.54 ± 0.54	21.74 ± 0.56 ^b^	31.52 ± 0.83	45	112.46 ± 5.96
	TT	15	4.94 ± 0.39 ^b^	67.61 ± 1.40	23.39 ± 1.46 ^a^	29.92 ± 2.15	5	124.18 ± 15.04
	*p*-value		**0.043**	0.758	**0.015**	0.405		0.160
g.64617 C > T	CC	401	5.41 ± 0.10	66.31 ± 0.38	21.42 ± 0.39	32.52 ± 0.58	136	113.74 ± 4.12
	CT	166	5.51 ± 0.14	66.71 ± 0.50	21.83 ± 0.53	31.92 ± 0.77	54	115.05 ± 5.42
	TT	21	5.89 ± 0.33	66.47 ± 1.20	19.71 ± 1.25	29.70 ± 1.84	6	114.24 ± 13.97
	*p*-value		0.474	0.521	0.222	0.385		0.995
g.67836 G > A	AA	12	5.46 ± 0.43	66.65 ± 1.57	24.59 ± 1.61 ^a^	32.16 ± 2.39	3	90.46 ± 20.00
	GA	158	5.42 ± 0.14	65.91 ± 0.52	21.40 ± 0.53 ^b^	33.36 ± 0.79	40	115.51 ± 6.06
	GG	418	5.48 ± 0.10	66.61 ± 0.38	21.23 ± 0.39 ^b^	31.77 ± 0.57	153	114.25 ± 3.97
	*p*-value		0.773	0.348	**0.000**	0.096		0.551
g.68017 G > A	AA	177	5.50 ± 0.14	67.03 ± 0.49	21.62 ± 0.53	31.58 ± 0.75	64	121.10 ± 5.18
	GA	269	5.46 ± 0.12	66.37 ± 0.42	21.52 ± 0.44	32.32 ± 0.65	89	115.82 ± 473
	GG	145	5.43 ± 0.14	65.65 ± 0.51	21.10 ± 0.51	32.93 ± 0.79	45	106.82 ± 5.38
	*p*-value		0.277	0.134	0.649	0.514		0.098

Bold values indicate *p* < 0.05; data in the same column with different lowercase letters on the shoulders indicate significant differences (*p* < 0.05). *p* is derived from the general linear mixed models (GLMMs). HCW: hot carcass weight; REA: rib eye area; WBSF: Warner–Bratzler shear force; DLR: drop loss rate; CLR: cook loss rate.

**Table 5:** Association between the presence and absence of *ACACB* haplotypes and carcass and meat quality traits (mean ± SE) ^a^ in yak.

Trait (Unit)^2^	Haplotype	n		Single-Haplotype Model		*p*	Multi-Haplotype Model			*p*
		Present	Absent	Present	Absent		Other Haplotypes in Model	Present	Absent	
WBSF (kg)	H1	245	277	5.33 ± 0.11	5.43 ± 0.12	0.420	H2, H5, H6	5.30 ± 0.16	5.43 ± 0.14	0.358
	H2	89	433	5.56 ± 0.17	5.34 ± 0.10	0.186	H5, H6	5.49 ± 0.19	5.30 ± 0.12	0.220
	H3	112	410	5.34 ± 0.15	5.38 ± 0.10	0.797	H2, H5, H6	5.37 ± 0.20	5.39 ± 0.13	0.896
	H4	156	366	5.33 ± 0.14	5.39 ± 0.10	0.639	H2, H5, H6	5.35 ± 0.18	5.40 ± 0.13	0.743
	H5	137	385	5.31 ± 0.14	5.58 ± 0.10	**0.049**	H2, H6	5.25 ± 0.17	5.35 ± 0.13	**0.051**
	H6	104	418	5.03 ± 0.16	5.45 ± 0.10	**0.006**	H2, H5	5.20 ± 0.18	5.59 ± 0.12	**0.011**
	H7	40	482	5.28 ± 0.24	5.41 ± 0.10	0.576	H2, H5, H6	5.17 ± 0.27	5.39 ± 0.13	0.336
	H8	72	450	5.55 ± 0.18	5.38 ± 0.10	0.322	H2, H5, H6	5.64 ± 0.21	5.37 ± 0.13	0.127
CLR (%)	H1	245	277	66.08 ± 0.43	66.72 ± 0.46	0.171	H3, H4, H7	65.60 ± 0.65	66.36 ± 0.60	0.120
	H2	89	433	66.75 ± 0.65	66.30 ± 0.38	0.468	H1, H3, H4, H7	66.50 ± 0.90	66.01 ± 0.58	0.449
	H3	112	410	67.60 ± 0.56	65.96 ± 0.39	**0.004**	H1, H4, H7	66.54 ± 0.72	65.31 ± 0.55	**0.020**
	H4	156	366	65.80 ± 0.52	66.58 ± 0.40	0.129	H1, H3, H7	65.64 ± 0.69	66.32 ± 0.57	0.200
	H5	137	385	66.50 ± 0.55	66.32 ± 0.39	0.737	H1, H3, H4, H7	65.88 ± 0.82	65.98 ± 0.57	0.868
	H6	104	418	66.23 ± 0.60	66.39 ± 0.39	0.777	H1, H3, H4, H7	65.57 ± 0.85	65.99 ± 0.57	0.505
	H7	40	482	65.00 ± 0.90	66.44 ± 0.37	0.097	H1, H3, H4	65.28 ± 0.92	66.68 ± 0.42	0.107
	H8	72	450	66.11 ± 0.68	66.41 ± 0.39	0.663	H1, H3, H4, H7	65.22 ± 0.88	66.01 ± 0.57	0.259
DLR (%)	H1	245	277	21.45 ± 0.42	21.11 ± 0.45	0.476				
	H2	89	433	21.79 ± 0.65	21.22 ± 0.38	0.356				
	H3	112	410	21.25 ± 0.56	21.32 ± 0.40	0.909				
	H4	156	366	21.55 ± 0.52	21.20 ± 0.40	0.489				
	H5	137	385	21.65 ± 0.55	21.20 ± 0.39	0.385				
	H6	104	418	21.16 ± 0.60	21.33 ± 0.39	0.762				
	H7	40	482	22.15 ± 0.90	21.25 ± 0.37	0.298				
	H8	72	450	19.96 ± 0.68	21.53 ± 0.38	**0.019**				
REA (cm^2^)	H1	245	277	32.48 ± 0.65	31.79 ± 0.70	0.336				
	H2	89	433	31.92 ± 0.99	32.22 ± 0.58	0.754				
	H3	112	410	31.55 ± 0.87	32.38 ± 0.61	0.339				
	H4	156	366	33.37 ± 0.80	31.72 ± 0.61	**0.034**				
	H5	137	385	31.57 ± 0.84	32.35 ± 0.60	0.332				
	H6	104	418	32.04 ± 0.92	32.21 ± 0.59	0.842				
	H7	40	482	32.62 ± 1.38	32.15 ± 0.57	0.725				
	H8	72	450	31.63 ± 1.04	32.27 ± 0.59	0.536				
HCW (kg)	H1	93	86	102.43 ± 3.85	110.05 ± 4.15	0.094				
	H2	30	149	105.92 ± 6.38	105.79 ± 3.39	0.983				
	H3	36	143	109.55 ± 5.45	104.70 ± 3.55	0.387				
	H4	41	138	109.94 ± 5.20	104.42 ± 3.57	0.303				
	H5	43	136	111.64 ± 5.26	104.03 ± 3.52	0.155				
	H6	41	138	106.97 ± 5.35	105.47 ± 5.32	0.781				
	H7	12	167	108.63 ± 9.03	105.58 ± 3.38	0.736				
	H8	27	152	106.36 ± 6.38	105.70 ± 3.45	0.918				

Bold values indicate *p* < 0.05. ^a^ Estimated marginal means and standard errors (SE); *p* is derived from the general linear mixed models (GLMMs). HCW: hot carcass weight; REA: rib eye area; WBSF: Warner–Bratzler shear force; DLR: drop loss rate; CLR: cook loss rate.

**Table 6:** Association between *ACACB* haplotype copy numbers and carcass and meat quality traits (mean ± SE) ^a^ in yak.

Trait (Unit)^2^	Haplotype	n			Single-Haplotype Model			*p*	Multi-Haplotype Model				*p*
		Absent	One Copy Present	Two Copy Present	Absent	One Copy Present	Two Copy Present		Other Haplotypes in Model	Absent	One Copy Present	Two Copy Present	
WBSF (kg)	H1	277	204	41	5.43 ± 0.12	5.30 ± 0.12	5.46 ± 0.23	0.570	H2, H5, H6	5.75 ± 0.32	5.62 ± 0.36	5.80 ± 0.42	0.539
	H2	433	83	6	5.34 ± 0.10	5.47 ± 0.18	6.56 ± 0.57	0.077	H5, H6	5.31 ± 0.24	5.43 ± 0.30	6.53 ± 0.62	0.085
	H3	410	103	9	5.37 ± 0.10	5.37 ± 0.15	4.86 ± 0.48	0.549	H2, H5, H6	5.74 ± 0.32	5.76 ± 0.37	5.27 ± 0.58	0.587
	H4	366	145	11	5.39 ± 0.10	5.28 ± 0.14	5.90 ± 0.43	0.322	H2, H5, H6	5.77 ± 0.32	5.69 ± 0.36	6.33 ± 0.55	0.316
	H5	385	123	14	5.30 ± 0.10	5.63 ± 0.15	5.18 ± 0.38	0.075	H2, H6	5.71 ± 0.28 ^b^	6.04 ± 0.31 ^a^	5.53 ± 0.47 ^b^	**0.046**
	H6	418	98	6	5.52 ± 0.10 ^a^	5.44 ± 0.16 ^b^	4.99 ± 0.57 ^b^	**0.014**	H2, H5	6.02 ± 0.25 ^a^	5.41 ± 0.29 ^b^	5.84 ± 0.62 ^b^	**0.021**
	H8	450	70	2	5.34 ± 0.10	5.54 ± 0.18	5.93 ± 0.98	0.450	H2, H5, H6	5.78 ± 0.32	6.06 ± 0.37	6.42 ± 1.03	0.249
CLR (%)	H1	277	204	41	66.72 ± 0.46	66.92 ± 0.45	66.85 ± 0.86	0.232					
	H2	433	83	6	66.30 ± 0.38	66.84 ± 0.67	65.58 ± 2.19	0.658					
	H3	410	103	9	66.00 ± 0.39 ^bc^	67.45 ± 0.58 ^b^	69.20 ± 1.80 ^a^	**0.007**					
	H4	366	145	11	66.58 ± 0.40	65.81 ± 0.53	65.78 ± 1.63	0.316					
	H5	385	123	14	66.32 ± 0.39	66.36 ± 0.57	67.75 ± 1.45	0.611					
	H6	418	98	6	66.39 ± 0.39	66.25 ± 0.62	65.87 ± 2.18	0.947					
	H8	450	70	2	66.41 ± 0.39	66.06 ± 0.69	67.88 ± 3.76	0.811					
DLR (%)	H1	277	204	41	21.12 ± 0.45 ^ab^	21.84 ± 0.45 ^a^	19.52 ± 0.86 ^b^	**0.028**	H8	17.70 ± 1.29 ^b^	18.17 ± 1.32 ^a^	15.71 ± 1.52 ^ab^	**0.023**
	H2	433	83	6	21.23 ± 0.38	21.95 ± 0.67	19.74 ± 2.18	0.402	H1, H8	17.19 ± 1.31	17.60 ± 1.46	15.45 ± 2.54	0.575
	H3	410	103	9	21.31 ± 0.40	21.27 ± 0.58	21.03 ± 1.81	0.985	H1, H8	17.22 ± 1.31	16.91 ± 1.41	16.71 ± 2.22	0.842
	H4	366	145	11	21.21 ± 0.40	21.47 ± 0.53	22.70 ± 1.62	0.596	H1, H8	17.21 ± 1.31	17.19 ± 1.41	18.41 ± 2.10	0.752
	H5	385	123	14	21.20 ± 0.39	21.64 ± 0.57	21.74 ± 1.45	0.684	H1, H8	17.20 ± 1.31	17.35 ± 1.43	17.55 ± 1.97	0.947
	H6	418	98	6	21.33 ± 0.39	21.16 ± 0.62	21.03 ± 2.17	0.954	H1, H8	17.19 ± 1.31	16.82 ± 1.44	16.47 ± 2.54	0.797
	H8	450	70	2	21.52 ± 0.38 ^a^	20.18 ± 0.68 ^b^	11.55 ± 3.71 ^c^	**0.004**	H1	21.00 ± 0.42 ^a^	19.58 ± 0.74 ^b^	11.00 ± 3.70 ^c^	**0.004**
REA (cm^2^)	H1	277	204	41	31.79 ± 0.70	32.51 ± 0.70	32.34 ± 1.32	0.625					
	H2	433	83	6	32.22 ± 0.58	32.03 ± 1.02	30.54 ± 3.35	0.867					
	H3	410	103	9	32.30 ± 0.61	31.91 ± 0.88	26.60 ± 2.76	0.107					
	H4	366	145	11	31.72 ± 0.61	33.36 ± 0.81	33.50 ± 2.48	0.105					
	H5	385	123	14	32.35 ± 0.60	31.66 ± 0.88	30.78 ± 2.22	0.580					
	H6	418	98	6	32.21 ± 0.59	32.05 ± 0.95	31.81 ± 3.34	0.978					
	H8	450	70	2	32.27 ± 0.59	31.60 ± 1.06	32.83 ± 0.75	0.807					
HCW (kg)	H1	83	77	19	109.40 ± 4.10 ^a^	105.99 ± 4.05 ^ab^	86.68 ± 7.32 ^c^	**0.011**	H4	101.72 ± 6.56 ^a^	97.79 ± 7.05 ^ab^	79.17 ± 9.54 ^c^	**0.014**
	H2	149	28	2	105.79 ± 3.40	106.24 ± 6.56	101.36 ± 21.51	0.975	H1, H4	92.72 ± 6.79	88.84 ± 9.38	82.63 ± 22.38	0.755
	H4	138	38	3	104.44 ± 3.55	112.08 ± 5.34	83.02 ± 17.39	0.160	H1	100.27 ± 3.82	104.73 ± 5.81	73.69 ± 17.38	0.197
	H5	138	38	3	103.92 ± 3.53	110.40 ± 5.62	119.53 ± 13.63	0.300	H1, H4	92.78 ± 6.78	95.27 ± 9.03	105.99 ± 15.57	0.625
	H6	138	33	167	105.44 ± 3.53	106.27 ± 5.57	115.20 ± 18.26	0.861	H1, H4	92.91 ± 6.79	90.21 ± 9.14	96.25 ± 19.65	0.880
	H8	153	25	1	105.64 ± 3.46	107.63 ± 6.60	97.84 ± 30.52	0.945	H1, H_4_	92.75 ± 6.79	89.13 ± 9.89	77.77 ± 30.82	0.784

Bold values indicate *p* < 0.05; data in the same column with different lowercase letters on the shoulders indicate significant differences (*p* < 0.05). ^a^ Estimated marginal means and standard errors (SE); *p* is derived from the general linear mixed models (GLMMs). HCW: hot carcass weight; REA: rib eye area; WBSF: Warner–Bratzler shear force; DLR: drop loss rate; CLR: cook loss rate.

**Table 7:** Association between *ACACB* diplotypes and carcass and meat quality traits (Mean ± SE) ^a^ in yak.

Diplotypes	Meat Quality					Carcass Quality	
	n	WBSF (kg)	CLR (%)	DLR (%)	REA (cm^2^)	n	HCW (kg)
H1H1	41	5.45 ± 0.22	66.59 ± 0.92	19.65 ± 0.91	32.15 ± 1.47	19	87.99 ± 8.21
H1H2	32	5.16 ± 0.25	67.17 ± 1.05	22.29 ± 1.04	30.76 ± 1.67	12	94.52 ± 10.07
H1H3	43	5.29 ± 0.21	66.91 ± 0.88	23.09 ± 0.87	33.39 ± 1.41	20	114.38 ± 7.44
H1H4	45	5.12 ± 0.21	65.12 ± 0.89	22.19 ± 0.87	33.50 ± 1.41	15	110.03 ± 8.76
H1H5	34	5.77 ± 0.24	65.12 ± 1.01	20.98 ± 1.00	31.01 ± 1.62	11	111.14 ± 10.32
H1H6	23	5.05 ± 0.29	66.22 ± 1.22	21.16 ± 1.21	32.46 ± 1.95	11	97.36 ± 9.97
H4H5	24	5.65 ± 0.29	65.82 ± 1.22	21.70 ± 1.20	32.31 ± 1.94	6	112.55 ± 13.42
H4H6	18	4.72 ± 0.33	65.31 ± 1.38	20.57 ± 1.36	33.29 ± 2.20	2	83.57 ± 22.65
H5H6	23	5.30 ± 0.30	66.17 ± 1.24	21.66 ± 1.23	30.74 ± 1.99	10	122.13 ± 10.60
H7H8	32	5.16 ± 0.25	67.17 ± 1.05	22.29 ± 1.04	30.76 ± 1.67	12	94.52 ± 10.07
*p*-value		0.146	0.716	0.220	0.839		0.095

**Table 8:** Primer sequence information for the three regions of yak *ACACB*.

Gene	Region	Primer Sequence (5′–3′)	Amplicon Size (bp)	Annealing Temperature (°C)
*ACACB*	Exon 37–intron 37	F: AAAATCTTCTTCCTCCCTG R: CGTGTATCTGTGCCGTCTA	455	60
*ACACB*	Exon 46–intron 46	F: ACGGTGGCTGCCTTGCTTT R: ATGCTGGACGCTGGTTTCA	362	60
*ACACB*	Intron 47	F: TCCCAGAGCACTTTACTT R: ATACCCGTCATCACCAT	790	60

## Data Availability

The data presented in this study are available on request from the corresponding author.

## References

[1] Yang S., Liu J., Gu Z., Liu P., Lan Q. (2022). Physiological and Metabolic Adaptation to Heat Stress at Different Altitudes in Yaks. Metabolites.

[2] Wang L., Li J., Teng S., Zhang W., Purslow P., Zhang R. (2022). Changes in Collagen Properties and Cathepsin Activity of Beef M. Semitendinosus by the Application of Ultrasound During Post-Mortem Aging. Meat Sci..

[3] Liu P., Ding L., Zhou Y., Jing X., Degen A.A. (2019). Behavioural Characteristics of Yaks Grazing Summer and Winter Pastures on the Qinghai-Tibetan Plateau. Appl. Anim. Behav. Sci..

[4] Guo X., Long R., Kreuzer M., Ding L., Shang Z., Zhang Y., Yang Y. (2014). Importance of Functional Ingredients in Yak Milk-Derived Food on Health of Tibetan Nomads Living under High-Altitude Stress: A Review. Crit. Rev. Food Sci. Nutr..

[5] Wen W., Luo X., Xia B., Guan J., Nie Y., Li L., Duan J., Suman S.P., Sun Q. (2015). Post-Mortem Oxidative Stability of Three Yak (Bos Grunniens) Muscles as Influenced by Animal Age. Meat Sci..

[6] Hu J., Gao X., Shi B., Chen H., Zhao Z., Wang J., Liu X., Li S., Luo Y. (2021). Sequence and Haplotypes of Ankyrin 1 Gene (Ank1) and Their Association with Carcass and Meat Quality Traits in Yak. Mamm. Genome.

[7] Picard B., Gagaoua M., Micol D., Cassar-Malek I., Hocquette J.F., Terlouw C.E. (2014). Inverse Relationships between Biomarkers and Beef Tenderness According to Contractile and Metabolic Properties of the Muscle. J. Agric. Food Chem..

[8] Sakowski T., Grodkowski G., Golebiewski M., Slosarz J., Kostusiak P., Solarczyk P., Puppel K. (2022). Genetic and Environmental Determinants of Beef Quality-a Review. Front Vet. Sci..

[9] Yin H., Zhang S., Gilbert E.R., Siegel P.B., Zhu Q., Wong E.A. (2014). Expression Profiles of Muscle Genes in Postnatal Skeletal Muscle in Lines of Chickens Divergently Selected for High and Low Body Weight. Poult. Sci..

[10] Ekine-Dzivenu C., Vinsky M., Basarab J.A., Aalhus J.L., Dugan M.E.R., Li C. (2017). Phenotypic and Genetic Correlations of Fatty Acid Composition in Subcutaneous Adipose Tissue with Carcass Merit and Meat Tenderness Traits in Canadian Beef Cattle. J. Anim. Sci..

[11] Gagaoua M., Bonnet M., Picard B. (2020). Protein Array-Based Approach to Evaluate Biomarkers of Beef Tenderness and Marbling in Cows: Understanding of the Underlying Mechanisms and Prediction. Foods.

[12] Pewan S.B., Otto J.R., Huerlimann R., Budd A.M., Mwangi F.W., Edmunds R.C., Holman B.W.B., Henry M.L.E., Kinobe R.T., Adegboye O.A. (2020). Genetics of Omega-3 Long-Chain Polyunsaturated Fatty Acid Metabolism and Meat Eating Quality in Tattykeel Australian White Lambs. Genes.

[13] Wen Y., Liu H., Liu K., Cao H., Mao H., Dong X., Yin Z. (2020). Analysis of the Physical Meat Quality in Partridge (Alectoris Chukar) and Its Relationship with Intramuscular Fat. Poult. Sci..

[14] Qiu F., Xie L., Ma J.E., Luo W., Zhang L., Chao Z., Chen S., Nie Q., Lin Z., Zhang X. (2017). Lower Expression of Slc27a1 Enhances Intramuscular Fat Deposition in Chicken Via Down-Regulated Fatty Acid Oxidation Mediated by Cpt1a. Front. Physiol..

[15] Guo Q., Kong X., Hu C., Zhou B., Wang C., Shen Q.W. (2019). Fatty Acid Content, Flavor Compounds, and Sensory Quality of Pork Loin as Affected by Dietary Supplementation with L-Arginine and Glutamic Acid. J. Food Sci..

[16] Liu S., Huang J., Wang X., Ma Y. (2020). Transcription Factors Regulate Adipocyte Differentiation in Beef Cattle. Anim. Genet..

[17] Boudon S., Henry-Berger J., Cassar-Malek I. (2020). Aggregation of Omic Data and Secretome Prediction Enable the Discovery of Candidate Plasma Biomarkers for Beef Tenderness. Int. J. Mol. Sci..

[18] Chung H., Shin S., Chung E. (2014). Effects of Genetic Variants for the Bovine Calpain Gene on Meat Tenderness. Mol. Biol. Rep..

[19] Marty A., Amigues Y., Servin B., Renand G., Leveziel H., Rocha D. (2010). Genetic Variability and Linkage Disequilibrium Patterns in the Bovine Dnaja1 Gene. Mol. Biotechnol..

[20] Goszczynski D.E., Papaleo-Mazzucco J., Ripoli M.V., Villarreal E.L., Rogberg-Munoz A., Mezzadra C.A., Melucci L.M., Giovambattista G. (2017). Genetic Variation in Fabp4 and Evaluation of Its Effects on Beef Cattle Fat Content. Anim. Biotechnol..

[21] Cesar A.S., Regitano L.C., Koltes J.E., Fritz-Waters E.R., Lanna D.P., Gasparin G., Mourao G.B., Oliveira P.S., Reecy J.M., Coutinho L.L. (2015). Putative Regulatory Factors Associated with Intramuscular Fat Content. PLoS ONE.

[22] Foldi J., Marczyk M., Gunasekharan V., Qing T., Sehgal R., Shan N.L., Muthusamy V., Umlau S., Surovtseva Y.V., Kibbey R. (2022). Abstract P5-17-01: Targeting Acetyl-Coa Carboxylase in Pre-Clinical Breast Cancer Models. Cancer Res..

[23] Parekh N., Chandran U., Bandera E.V. (2012). Obesity in Cancer Survival. Annu. Rev. Nutr..

[24] Zu X., Zhong J., Luo D., Tan J., Zhang Q., Wu Y., Liu J., Cao R., Wen G., Cao D. (2013). Chemical Genetics of Acetyl-Coa Carboxylases. Molecules.

[25] Zhang S., Kim K.H. (1996). Acetyl-Coa Carboxylase Is Essential for Nutrient-Induced Insulin Secretion. Biochem. Biophys. Res. Commun..

[26] Zain M., Awan F.R., Najam S.S., Islam M., Khan A.R., Bilal A., Bellili N., Marre M., Roussel R., Fumeron F. (2017). Association of Acacb Gene Polymorphism (Rs2268388, G > a) with Type 2 Diabetes and End Stage Renal Disease in Pakistani Punjabi Population. Meta Gene.

[27] Harwood H.J. (2005). Treating the Metabolic Syndrome: Acetyl-Coa Carboxylase Inhibition. Expert Opin. Ther. Targets.

[28] An L., Jiang H., Tang R.N. (2015). The Acacb Gene Rs2268388 Polymorphism Is Associated with Nephropathy in Caucasian Patients with Diabetes: A Meta-Analysis. Ren. Fail..

[29] Riancho J.A., Vazquez L., Garcia-Perez M.A., Sainz J., Olmos J.M., Hernandez J.L., Perez-Lopez J., Amado J.A., Zarrabeitia M.T., Cano A. (2011). Association of Acacb Polymorphisms with Obesity and Diabetes. Mol. Genet. Metab..

[30] Costa A.S.H., Costa P., Alves S.P., Alfaia C.M., Prates J.A., Vleck V., Cassar-Malek I., Hocquette J.F., Bessa R.J. (2018). Does Growth Path Influence Beef Lipid Deposition and Fatty Acid Composition?. PLoS ONE.

[31] Lee A.K., Kyriakou T., Weston A.J., O’Dell S.D. (2010). Functional Single-Nucleotide Polymorphism in Acetyl-Coa Carboxylase Acacb Gene Promoter. DNA Cell Biol..

[32] Shi Y.Y., He L. (2005). Shesis, a Powerful Software Platform for Analyses of Linkage Disequilibrium, Haplotype Construction, and Genetic Association at Polymorphism Loci. Cell Res..

[33] Raza S.H.A., Gui L., Khan R., Schreurs N.M., Xiaoyu W., Wu S., Mei C., Wang L., Ma X., Wei D. (2018). Association between Fasn Gene Polymorphisms Ultrasound Carcass Traits and Intramuscular Fat in Qinchuan Cattle. Gene.

[34] Shergojry S.A., Verma A., Ghani M., Gupta I.D., Mir N.A. (2023). Identification of Genetic Polymorphism of the Mbl2 Gene and Its Association with Clinical Mastitis in Murrah Buffaloes. J. Genet..

[35] Li Z., Zhang Z., He Z., Tang W., Li T., Zeng Z., He L., Shi Y. (2009). A Partition-Ligation-Combination-Subdivision Em Algorithm for Haplotype Inference with Multiallelic Markers: Update of the Shesis (http://Analysis.Bio-X.Cn). Cell Res..

[36] Abu-Elheiga L., Jayakumar A., Baldini A., Chirala S.S., Wakil S.J. (1995). Human Acetyl-Coa Carboxylase: Characterization, Molecular Cloning, and Evidence for Two Isoforms. Proc. Natl. Acad. Sci. USA.

[37] Tong L. (2005). Acetyl-Coenzyme a Carboxylase: Crucial Metabolic Enzyme and Attractive Target for Drug Discovery. Cell. Mol. Life Sci..

[38] Ma L., Mondal A.K., Murea M., Sharma N.K., Tönjes A., Langberg K.A., Das S.K., Franks P.W., Kovacs P., Antinozzi P.A. (2011). The Effect of Acacb Cis-Variants on Gene Expression and Metabolic Traits. PLoS ONE.

[39] Maeda S., Kobayashi M.A., Araki S.I., Babazono T., Freedman B.I., Bostrom M.A., Cooke J.N., Toyoda M., Umezono T., Tarnow L. (2010). A Single Nucleotide Polymorphism within the Acetyl-Coenzyme a Carboxylase Beta Gene Is Associated with Proteinuria in Patients with Type 2 Diabetes. PLoS Genet..

[40] Tang S.C., Leung V.T., Chan L.Y., Wong S.S., Chu D.W., Leung J.C., Ho Y.W., Lai K.N., Ma L., Elbein S.C. (2010). The Acetyl-Coenzyme a Carboxylase Beta (Acacb) Gene Is Associated with Nephropathy in Chinese Patients with Type 2 Diabetes. Nephrol. Dial. Transpl..

[41] Shah V.N., Cheema B.S., Sharma R., Khullar M., Kohli H.S., Ahluwalia T.S., Mohan V., Bhansali A. (2013). Acacbeta Gene (Rs2268388) and Agtr1 Gene (Rs5186) Polymorphism and the Risk of Nephropathy in Asian Indian Patients with Type 2 Diabetes. Mol. Cell. Biochem..

[42] Han B., Liang W., Liu L., Li Y., Sun D. (2018). Genetic Association of the Acacb Gene with Milk Yield and Composition Traits in Dairy Cattle. Anim. Genet..

[43] Neil C.R., Fairbrother W.G. (2019). Intronic RNA: Ad ’junk’ Mediator of Post-Transcriptional Gene Regulation. Biochim. Biophys. Acta Gene. Regul. Mech..

[44] Yeoh G., Barton S., Kaestner K. (2011). The International Journal of Biochemistry & Cell Biology. Preface. Int. J. Biochem. Cell. Biol..

[45] Wiik M.U., Evans T.J., Belhadj S., Bolton K.A., Dymerska D., Jagmohan-Changur S., Capellá G., Kurzawski G., Wijnen J.T., Valle L. (2021). A Genetic Variant in Telomerase Reverse Transcriptase (Tert) Modifies Cancer Risk in Lynch Syndrome Patients Harbouring Pathogenic Msh2 Variants. Sci. Rep..

[46] McNamara J.W., Schuckman M., Becker R.C., Sadayappan S. (2020). A Novel Homozygous Intronic Variant in Tnnt2 Associates with Feline Cardiomyopathy. Front. Physiol..

[47] Shaul O. (2017). How Introns Enhance Gene Expression. Int. J. Biochem. Cell. Biol..

[48] Nott A., Meislin S.H., Moore M.J. (2003). A Quantitative Analysis of Intron Effects on Mammalian Gene Expression. RNA.

[49] Chorev M., Carmel L. (2012). The Function of Introns. Front. Genet..

[50] Gao X., Shi B., Shi X., Zuo Z., Zhao Z., Wang J., Liu X., Luo Y., Hu J. (2020). Variations in the Diacylglycerol Acyltransferase-1 (Dgat1) and Its Association with Meat Tenderness in Gannan Yaks (*Bos grunniens*). Ital. J. Anim. Sci..

[51] Angiolillo A., Amills M., Urrutia B., Doménech A., Sastre Y., Badaoui B., Jordana J. (2007). Identification of a Single Nucleotide Polymorphism at Intron 16 of the Caprine Acyl-Coenzyme A: Diacylglycerol Acyltransferase 1 (Dgat1) Gene. J. Dairy Res..

[52] Wang T., Shi X., Liu Z., Ren W., Wang X., Huang B., Kou X., Liang H., Wang C., Chai W. (2022). A Novel a > G Polymorphism in the Intron 1 of Lcorl Gene Is Significantly Associated with Hide Weight and Body Size in Dezhou Donkey. Animals.

[53] Wang Y., Li H., Gu Z.L., Zhao J.G., Wang Q.G., Wang Y.X. (2004). Correlation Analysis between Single Nucleotide Polymorphism of the Leptin Receptor Intron 8 and Fatness Traits in Chickens. Yi Chuan Xue Bao = Acta Genet. Sin..

[54] Grochowska E., Borys B., Mroczkowski S. (2020). Effects of Intronic Snps in the Myostatin Gene on Growth and Carcass Traits in Colored Polish Merino Sheep. Genes.

[55] Wang W., Wu Z., Dai Z., Yang Y., Wang J., Wu G. (2013). Glycine Metabolism in Animals and Humans: Implications for Nutrition and Health. Amino Acids.

[56] Zhong Y., Yan Z., Song B., Zheng C., Duan Y., Kong X., Deng J., Li F. (2021). Dietary Supplementation with Betaine or Glycine Improves the Carcass Trait, Meat Quality and Lipid Metabolism of Finishing Mini-Pigs. Anim. Nutr..

[57] Snyder M.W., Adey A., Kitzman J.O., Shendure J. (2015). Haplotype-Resolved Genome Sequencing: Experimental Methods and Applications. Nat. Rev. Genet..

[58] Boichard D., Ducrocq V., Croiseau P., Fritz S. (2016). Genomic Selection in Domestic Animals: Principles, Applications and Perspectives. C. R. Biol..

[59] Scheike T.H., Martinussen T., Silver J.D. (2010). Estimating Haplotype Effects for Survival Data. Biometrics.

[60] Sandrim V.C., Tanus-Santos J.E. (2007). Haplotype Analysis Can Provide Improved Clinical Information Than Single Genotype Analysis. Thromb. Res..

[61] An Q., Zhou H., Hu J., Luo Y., Hickford J.G.H. (2018). Sequence and Haplotypes Variation of the Ovine Uncoupling Protein-1 Gene (Ucp1) and Their Association with Growth and Carcass Traits in New Zealand Romney Lambs. Genes.

[62] Liu M., Peng J., Xu D.Q., Zheng R., Li F.E., Li J.L., Zuo B., Lei M.G., Xiong Y.Z., Deng C.Y. (2008). Association of Myf5 and Myod1 Gene Polymorphisms and Meat Quality Traits in Large White X Meishan F2 Pig Populations. Biochem. Genet..

[63] Honikel K.O. (1998). Reference Methods for the Assessment of Physical Characteristics of Meat. Meat Sci..

